# Timing and Sequence of Brain Activity in Top-Down Control of Visual-Spatial Attention

**DOI:** 10.1371/journal.pbio.0050012

**Published:** 2007-01-02

**Authors:** Tineke Grent-‘t-Jong, Marty G Woldorff

**Affiliations:** 1 Center for Cognitive Neuroscience, Duke University, Durham, North Carolina, United States of America; 2 Department of Psychopharmacology, University of Utrecht, Utrecht, The Netherlands; 3 Department of Psychiatry, Duke University, Durham, North Carolina, United States of America; National Institute of Mental Health-National Institutes of Health, United States of America

## Abstract

Recent brain imaging studies using functional magnetic resonance imaging (fMRI) have implicated a frontal-parietal network in the top-down control of attention. However, little is known about the timing and sequence of activations within this network. To investigate these timing questions, we used event-related electrical brain potentials (ERPs) and a specially designed visual-spatial attentional-cueing paradigm, which were applied as part of a multi-methodological approach that included a closely corresponding event-related fMRI study using an identical paradigm. In the first 400 ms post cue, attention-directing and control cues elicited similar general cue-processing activity, corresponding to the more lateral subregions of the frontal-parietal network identified with the fMRI. Following this, the attention-directing cues elicited a sustained negative-polarity brain wave that was absent for control cues. This activity could be linked to the more medial frontal–parietal subregions similarly identified in the fMRI as specifically involved in attentional orienting. Critically, both the scalp ERPs and the fMRI-seeded source modeling for this orienting-related activity indicated an earlier onset of frontal versus parietal contribution (∼400 versus ∼700 ms). This was then followed (∼800–900 ms) by pretarget biasing activity in the region-specific visual-sensory occipital cortex. These results indicate an activation sequence of key components of the attentional-control brain network, providing insight into their functional roles. More specifically, these results suggest that voluntary attentional orienting is initiated by medial portions of frontal cortex, which then recruit medial parietal areas. Together, these areas then implement biasing of region-specific visual-sensory cortex to facilitate the processing of upcoming visual stimuli.

## Author Summary

Attention is a fundamental cognitive function that allows us to focus neural resources on events or information in our environment that are most important or interesting to us at any given moment. Recent functional neuroimaging studies have indicated that a network of brain areas in frontal and parietal cortex is involved in directing our attention to specific locations in our visual field. However, little is known about the timing and sequence of activations within the various parts of this attentional control network, thus limiting our understanding of their functional roles. We extracted a more precise picture of the neural mechanisms of attentional control by combining two complementary methods of measuring cognitive brain activity: functional magnetic resonance imaging (fMRI) and electroencephalography (EEG). fMRI offers information on a millimeter scale about the locations of brain activity, whereas EEG offers temporal information on a scale of milliseconds. Our results indicate that visual-spatial attentional control is initiated in frontal brain areas, joined shortly afterwards by parietal involvement. Together, these brain areas then prepare relevant areas in the visual cortex for performing enhanced processing of visual input in the attended region of space.

## Abstract 3

A combination of methods reveals the timing and sequence of neural activation within the frontal-parietal network and provides a more precise picture of the mechanisms controlling visual attention in humans.

## Introduction

At every moment of our lives, we are deluged with sensory stimuli coming at us from multiple directions and through our various sensory modalities—much more information than we can fully process. The critical function of attention allows us at each moment to continuously select and extract the most important information from this flood of sensory inputs in order to provide those stimuli with fuller processing.

In the visual modality, if one covertly directs attention (i.e., without moving the eyes) to a location in the visual field, stimuli that occur in that location are discriminated or detected faster and/or better than those at other locations in the visual field [1,2]. Previous brain imaging and electrophysiological studies [3–8] have shown that this behavioral improvement is associated with increased evoked brain-activity responses in early visual sensory areas for stimuli that occur at the attended location. In addition, the directing of visual-spatial attention is associated with increased activity in a network of mainly dorsal frontal and parietal cortical brain areas [8–16]. It is thought that this frontal–parietal network may facilitate a biasing of the system in advance toward task-relevant information by enhancing baseline activity in feature-specific visual areas that will be processing the visual stimuli [7,17–22].

Whereas previous brain imaging studies of the top-down control of visual-spatial attention have helped delineate which brain areas exert control over stimulus processing, the various mechanisms by which this is implemented are still unclear. Apart from the finding of pre-target baseline shifts in visual areas that may facilitate upcoming target processing and that may be induced by top-down signals from the frontal–parietal control network, little is known about the timing and sequence of activations within this frontal-parietal network and their temporal relationship to such biasing. To date, no studies have addressed whether these areas act together in temporal concert, or whether there is some specific temporal sequence of the different components. Knowledge of the timing and sequence of activation within this network is important, because it could provide us with more specific information on functional specificity of these regions for attentional orienting, including which parts initiate and/or sustain the directed attention.

This incomplete understanding is partly due to the difficulty of comparing the various studies on top-down attentional control, which, in turn, is partly due to their different methodologies and different design structures. Various event-related potential (ERP) studies, for example, have looked at top-down control of attention [23–28]. These have typically used the event-related capabilities inherent in ERPs, applied to the cueing paradigm developed in behavioral studies in which an instructional attention-directing cue is followed by a target stimulus [1]. By time-locked averaging of the responses separately to the cues and to the targets, these studies have extracted the brain-wave activity triggered by each of them. However, none of these studies have reported the underlying neural sources of the cue-related ERP activity associated with attentional control, making their observations difficult to compare with hemodynamically based (i.e., blood-flow–based) functional imaging studies (e.g., those using positron emission tomography and functional MRI [fMRI]).

On the other hand, positron emission tomography and fMRI studies of attentional control have generally been structured rather differently from the ERP studies, and they have also had their limitations. First, and most critically, hemodynamically based fMRI signals are too sluggish to be able to reveal temporal aspects of the activations within the control network (e.g., timing differences between the frontal and parietal activations). Secondly, although ERP studies have generally used the relatively short cue-target intervals used in behavioral studies (∼1 s), event-related fMRI cueing studies have typically used much longer intervals (e.g., ∼4–10 s), mainly to be able to deal with the severe overlap of the hemodynamic response signals to the cues and the targets. Using such longer cue-target intervals, however, is likely to invoke different cognitive processes or subject strategies, adding to the difficulty of comparing to the electrophysiological data. Third, the responses extracted to the attention-directing cues have often included general processing of the cue (e.g., interpreting its meaning), and thus have not exclusively reflected attentional orienting. Alternatively, the analyses have used a contrast between directing attention toward one stimulus aspect versus toward another stimulus aspect, thereby subtracting out the overall orienting activity.

Considering the limitations of earlier fMRI studies, we previously designed a fast-rate event-related cueing fMRI study [22] with shorter cue-target intervals that are much more similar to those used in ERP and behavioral studies and with control-cue trials that controlled for general cue processing. In that study, participants received instructional cues telling them either to attend (attend cues) covertly to a location in the lower left or lower right visual field to detect a faint gray dot that might (or might not) be presented there, or cues telling them to not orient their attention (interpret cues) on that trial (see schematic trial structure in Figure 1). When the target did occur on the active-attend trials (attend-cue–plus–target trials), it could come either 900 ms (early) or 1,900 ms (late) randomly, but on some trials targets would not occur at all (attend-cue-only trials). Participants were also instructed to delay their response to any detected target until the onset of a “report” signal toward the end of the trial, thereby helping to minimize motor-preparation activity during the cue-target interval. This hierarchical design, with attend-cue-plus-target trials, attend-cue–only trials, and interpret-cue trials, allowed us to separate not only cue-related from target-related activity, but also to separate cue-induced attentional-orienting activity from more general cue-processing-related activity. Results from this fMRI study showed a clear dissociation between the more lateral subregions of the frontal-parietal network, which were activated similarly by both attend-cue and interpret-cue trials, and the more medial frontal and parietal subregions, which were more strongly activated by the attend cues. This pattern of results indicated that the lateral parts of the frontal-parietal network were involved in more general aspects of cue processing, such as cue-symbol interpretation and decisional processes based on that interpretation (i.e., whether to orient), whereas the more medial frontal-parietal network activity was more specifically related to the process of orienting of visual-spatial attention.

**Figure 1:**
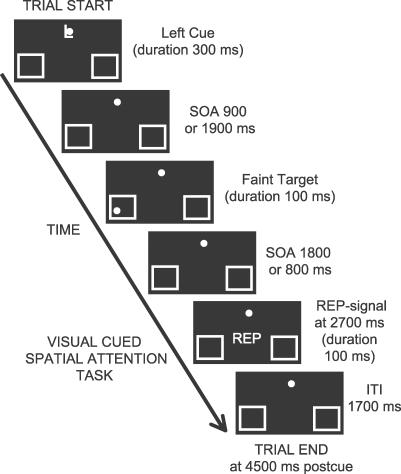
Example of an Attend-Left Cue–Plus–Target Trial A centrally presented left cue (letter L) instructed the participant to covertly attend to the lower left visual field box to detect an upcoming faint dot target that might be presented there. A target could appear either early or late (50% probability) following the cue and at the valid location only. An end-of-trial signal (the letters REP) presented at 2,700 ms post-cue signaled the participant to press a button to report if they had seen a target. Other trial types included attend-right cue–plus–target, attend-left cue–only (no target), attend-right cue–only (no target), interpret-cue (also no target), and NoStim trials (no cue nor target). SOA, stimulus onset asynchrony; ITI, interstimulus interval.

As noted above, the fMRI results alone cannot delineate the temporal characteristics of these activations. In the present study, we therefore recorded electroencephalography (EEG) and extracted ERPs using the same cued-attention paradigm (described above) as in our previous fMRI study [22].Although fMRI and EEG recording techniques measure different aspects of brain activity—and thus cannot be expected to have a perfect one-to-one relationship—various studies have shown close correlation between intracranially recorded local field potentials, including event-related, time-extended, local field potentials, and the event-related blood-oxygen-level–dependent (BOLD) signal used in fMRI [e.g., 29]. Moreover, numerous studies have also shown considerable correlation between scalp-recorded ERP components and fMRI BOLD responses, supporting the usefulness of comparing and combining the results gathered with the two methods. For example, successful attempts to combine ERP data with results from a comparable fMRI dataset have been described for early sensory ERP components, such as the visual P1 and N1 components [6,30–32], the face-specific N170 wave [33,34], the frontal target-detection–related N2 [35,36], the error-related negativity associated with incorrect target-detection responses [37,38], the auditory sensory N1 component, and the mismatch negativity elicited by auditory deviants in a stream of repeated stimuli [39–43]; as well as for later ERP components, such as the target-detection-related P3b [44–48], the language-related N400 component [49,50], and slow anticipation–related potentials, such as the contingent negative variation [51,52]. Accordingly, we hypothesized that the present combined ERP–fMRI source modeling approach would help elucidate the timing and sequence of activations within the frontal-parietal network, knowledge which would be useful in understanding the roles of these areas in visual-spatial attentional control.

## Results

### Frontal-Parietal Attentional Control Network Activity

Cue-triggered ERP waveforms that were time locked separately to the attend cues (collapsed across attend left and attend right) and to the interpret cues are shown in Figure 2A. For approximately the first 400 ms, traces for the attention-directing cues and interpret cues closely overlapped everywhere across the scalp, beginning to differentiate only after that time. By 600 ms, activation for interpret cues had essentially returned to baseline, whereas that evoked by attend cues during the rest of the cue-target interval took the form of a sustained, mostly bilateral negativity over frontal, central, and parietal scalp sites. In the difference waves, which were computed from the contrast between those attend and interpret cues (Figure 2B), the early evoked responses to both cue types (until 400 ms) thus essentially subtracted out, leaving the strong frontal-central-parietal negativity in the rest of the cue-target interval. Because these attentional-orienting activations over frontal, central, and parietal sites did not show any consistent contralaterality relative to the direction of attention, these data were collapsed across this dimension, and we have focused on the attend-cue versus the interpret-cue trials over these sites.

**Figure 2:**
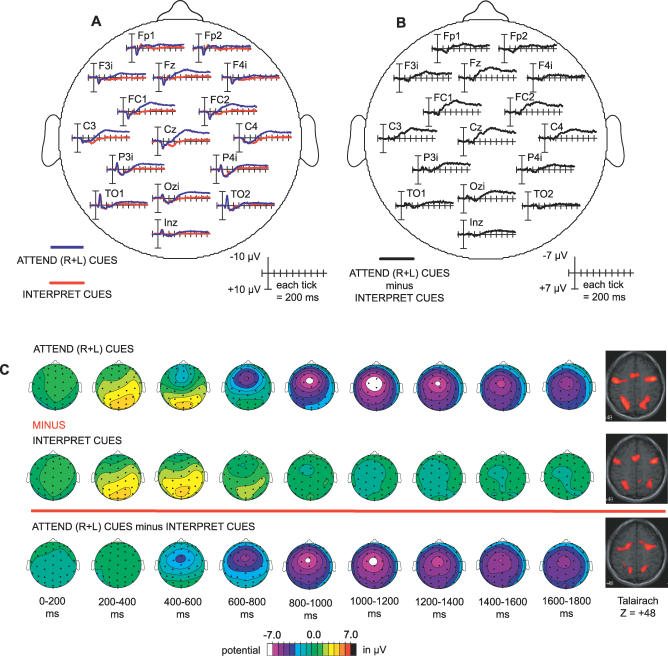
Cue-Related ERP and fMRI Responses (A) Grand average across participants (*n* = 13) of the ERP traces from 16 of the channels across the scalp of the cue-triggered responses to attend-cue trials (collapsed over right and left and also across cue-only and cue-plus–late-target trials), overlaid on the cue responses for interpret-cue trials, starting 200 ms before cue onset until 1,900 ms (which was the onset of a target on trials with a late target). (B) Grand-average (*n* = 13) ERP difference waves of the attend-cue responses minus interpret-cue responses from (A). (C) ERP topographic maps of these scalp-potential distributions, averaged over 200-ms windows, for attend cues, interpret cues, and their difference waves, starting at the onset of the cues and ending 100 ms before the time of a possible late target presentation. On the right are shown the corresponding fMRI activations (at Talairach height of *z* = +48) for the attend-cue–only and interpret-cue responses (corrected for overlap) and for the attend-cue–versus–interpret-cue contrasts observed in the identical conditions in the corresponding Woldorff et al. (2004) fMRI study [22].

The scalp potential topographic distribution maps across time for attend cues, interpret cues, and their difference waves are shown in Figure 2C. These maps, like the traces in Figure 2A and 2B, show clearly that the early ERP activity triggered by attend cues and interpret cues was very similar out to 400 ms post-cue. Thus, in the subtraction of these responses (bottom row, Figure 2C), this early activity subtracts away, leaving the sustained negativity beginning at around 400 ms and lasting throughout the entire cue-target interval. These maps also show that the early part of the attentional-orienting negativity (from around 400–800 ms post-cue) had a clear frontal distribution, whereas over the next few hundred milliseconds, the activity progressively spread posteriorly over the scalp to include central and parietal sites.

Repeated measures of variance (ANOVAs) applied to the ERP response amplitudes and computed for consecutive windows of 100 ms across the cue-target interval confirmed the presence of a frontal-central-parietal sustained negativity by showing highly significant main effects of ATTENTION (attend versus interpret cues) between 400–1,900 ms post-cue on all electrodes covering medial frontal, central, and parietal scalp sites (*F*[1,12] values between 9.3–78.1, all *p-*values < 0.01). However, the point in time after 400 ms in which this differential activity became significant varied systematically between frontal, central, and parietal scalp locations. More specifically, these attentional-orienting effects were significant over frontal sites beginning in the 400–500-ms window post-cue (*F*[1,12] values between 9.4–22.4, all *p-*values < 0.01) over central sites beginning at 500–600 ms post-cue (*F*[1,12] values between 9.3–36.0, all *p-*values < 0.01), and over parietal sites beginning at 700–800 ms post-cue (*F*[1,12] values between 9.9–27.1, all *p-*values < 0.01). At around 1,000–1,200 ms, the scalp distribution of the attentional-orienting difference wave activity stabilized, with no apparent further change in distribution across the rest of the cue-target interval out to 1,900 ms. This result suggests that the sustained activity for the rest of the cue-target interval stayed relatively constant in source configuration.

Because of the identical trial structures and contrasts between the ERP and fMRI studies, these ERP results could be mapped to the activations seen in the corresponding conditions in the fMRI study (Figure 2C, upper two rows). Therefore, the early ERP activity (before 400 ms) that was similarly triggered by both the interpret cues and attend cues (Figure 2C, upper two rows) presumably corresponds to the general cue-processing-related activity in such areas as visual sensory cortex and the lateral frontal-parietal network, which were similarly activated for the interpret and attend cues in the fMRI (Figure 2C, upper two rows, right panels). Similarly, the later sustained negativity remaining after the subtraction (Figure 2C, lowest row) that continued only for the attend cues would be expected to reflect the orienting-specific activation and to correspond to the more medial frontal-parietal areas activated mainly by the attend cues in the fMRI data (Figure 2C, bottom row, right).

With respect to the timing of activation within the different components of the orienting-specific activity in the medial frontal-parietal network, a number of observations pointed to an onset delay in the parietal areas compared to the frontal areas. First, as noted above, the attentional-orienting negativity, seen in the ERPs and the topographic plots, began with a more frontal distribution and started to become significant a few hundred milliseconds earlier over frontal scalp sites than over parietal ones. The earlier onset over frontal sites relative to more posterior locations can be seen particularly clearly in overlays of the ERP traces from the frontal and parietal scalp sites directly above the medial frontal-parietal network areas identified in the fMRI using the identical contrast, with onsets at about 400 and 650 ms, respectively (see Figure 3A, top, left and right hemisphere scalp ERPs).

**Figure 3:**
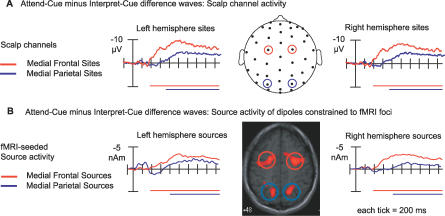
Timing of Activity in the Medial Frontal–Parietal Attentional-Orienting Network (A) Grand-average (*n* = 13) ERP traces selected from channels located at scalp sites above the medial frontal and medial parietal fMRI foci for the difference waves derived from the contrast of attend cues versus interpret cues. The traces for the frontal and parietal scalp are overlaid, showing the temporal delay for the parietal relative to the frontal scalp sites. Below the ERP traces, the horizontal colored bars indicate the windows in which the attentional-orienting activity across participants was significant at the frontal channels (red) and at the parietal channels (blue). (B) Overlay of the estimated source activity waveforms for the medial frontal and medial parietal sources, separately for each hemisphere, also showing the relative delay for the parietal relative to the frontal sources. These source activity waveforms resulted from source modeling of the grand-average (*n* = 13) ERP difference wave for attend cues versus interpret cues, using dipoles constrained to fMRI centroids of activity (obtained from Woldorff et. al 2004) [22]. Below the source activity waveforms, the horizontal colored bars represent the windows in which the attentional-orienting activity across participants was significant for the estimated frontal source (red) and parietal source (blue) activity waveforms.

Second, as another way to approach relative timing issues as well as to additionally relate these ERP activations to neuroanatomical brain regions, ERP source analysis was applied to the attentional-orienting difference wave activity using information from the analogous fMRI contrast. More specifically, two pairs of dipoles were placed at the centroids of the medial frontal and medial parietal fMRI activations, and iterative best-fit modeling of their orientation was applied to the ERP distributions (fMRI centroid locations are circled in Figure 3B, middle panel) (fMRI overlays; fMRI activation coordinates in Talairach space [53] were *x* = −23, *y* = −4, *z* = 46 [for the left medial frontal cortex, BA6]; *x* = 27, *y* = 1, *z* = 46 [for the right medial frontal cortex, BA6]; *x* = −18, *y* = − 58, *z* = 48 [for the left parietal (precuneus), BA7]; and *x* = 20, *y* = −57, *z* = 50 [for the right parietal (precuneus), BA7]). By using such an fMRI-seeded source modeling approach with location-constrained dipole sources, 95% of the variance for the observed ERP distribution was explained (residual variance [RV] = 5%) across the entire time period of the attentional-orienting effects (400–1,900 ms post-cue). Moreover, as was the case with the overlays of the frontal versus parietal scalp ERP traces, overlays of the analogous estimated source activity waveforms from these medial frontal and parietal sources (Figure 3B; left and right hemisphere source waveforms) also argue that the onset of the sustained frontal activity leads that of the sustained parietal activity by several hundred milliseconds, with onsets at around 400 and 700 ms, respectively

Third, source analyses were performed on the orienting-related difference-wave activity of individual participants, and the resultant estimated dipole strengths were statistically analyzed across paticipants. Residual variance found for the individual-subject dipole solutions, using fMRI-seeded sources with optimized best-fitting orientations, ranged from 3.8%–20.3%, calculated across the entire window of 400–1,900 ms. Consistent with the observed onset timing differences seen in the grand averages, the statistical analyses of the estimated dipole strengths revealed significant activity in the two medial frontal sources starting at 400 ms (400–1,900 ms, *t*-values between −3.2 and −7.1, all *p-*values < 0.01), whereas the later-onsetting sustained medial parietal sources started to become significantly activated several hundred milliseconds later (800–1,900 ms, *t*-values between −2.7 and −3.9, all *p-*values < 0.03). Note that these results for the source activity waveforms, which converge with the scalp ERP results described earlier indicating that the attentional orienting activity did not become significant over parietal scalp sites until after 700 ms, argue against a possible early parietal onset, even though the estimated grand-average left parietal source waveform might suggest a small amount of earlier transient parietal activity.

Fourth, if our conclusion of an earlier onset for frontal versus parietal activation is correct, then the frontal sources alone should provide a fairly good fit in a time window in which the frontal sources are active but the parietal ones are not yet active. Thus, to test this prediction, source analysis was applied to the window of 500–600 ms using just the two medial frontal sources alone, and, indeed, these explained 96% of the distribution (RV = 4%). In contrast, the two parietal sources alone had a considerably worse fit (RV = 13%) in this time period, leading to fits with orientations pointing very anteriorly.

Fifth, to provide additional converging evidence, we performed a free fit (i.e., both locations and orientations allowed to vary, not imposing any locational constraints from the fMRI results) of a bilaterally symmetric dipole pair during the 500–600 ms latency range. Again, consistent with our hypothesis that the frontal sources initiated the attentional orienting during this latency range, this free fit resulted in source locations in medial frontal cortex (RV = 4.4%), with Talairach coordinates fairly close to the medial frontal fMRI-derived sources (within 1 cm in all dimensions; left frontal [*x* = − 18, *y* = −8, *z* = 45] and right frontal cortex [*x * = 18, *y* = −8, *z* = 45]).

Sixth, additional analyses were performed to assess the relative contribution of the frontal and parietal sources over the entire time period of significant sustained activity. More specifically, the explained variance was computed in successive 200-ms windows between 500–1,900 ms using the frontal sources alone, the parietal sources alone, and the frontal and parietal sources combined (Figure 4A). The figure reaffirms what was noted above, namely that in the early windows, the two medial frontal sources alone provided a particularly good fit by themselves and considerably better than the parietal locations alone. Thereafter, the frontal sources by themselves still explained most of the variance in the data, but substantially less well. This could be taken as further evidence that additional sources had become active in the later part of the window. Indeed, as shown in Figure 4A, adding the two parietal sources in the later part of the time window substantially improved the fit, whereas they added little in the earlier time range.

**Figure 4:**
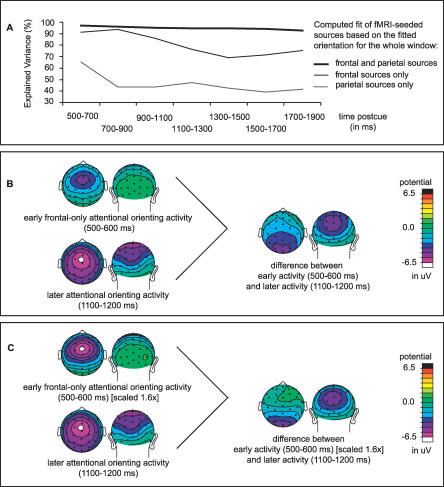
Evidence for Robustness of the Four-Source Frontal-Parietal Model of the Attentional Orienting Activity (A) Explained variance of three different source configurations (frontal only, parietal only, and both frontal and parietal) for the grand-average (*n* = 13) difference-wave activity between attend and interpret cue responses. These were computed across time (500–1,900 ms) in windows of 200 ms, using the locations and orientations of the sources from the initial fMRI-seeded solution. Note that in the earlier windows, the solution with only two frontal sources (thin black line) explains the variance in the data very well (and better than the solution with only two parietal sources). But later in time, both frontal and parietal sources (thick black line) are needed to explain the data. (B) Topographic distributions of the grand-average (*n* = 13) attentional-orienting activity at 500–600 ms (upper left map), at 1,100–1,200 ms (lower left map), and the calculated difference between these. Note that the residual activity after the subtraction has a clearly posterior parietal distribution, consistent with a model in which additional posterior sources activate later, rather than a model with only anterior sources that just become more strongly activated over time. (C) Same as (B) but with the attentional-orienting activity between 500–600 ms being scaled in amplitude first, before the subtraction from the activity during the distribution at the later window (i.e., amplitudes at all channels from the early activity were first multiplied by a factor 1.6 to approximately match the amplitude of the midfrontal activity in the later window between 1,100–1,200 ms). Note that the frontal activity is now even more subtracted out, but there still remains clear activity with a posterior parietal maximum.

And lastly, we performed an additional analysis of the attentional-orienting activity to provide further evidence for the addition of two parietal sources that onset later, in contrast to a possible alternative model in which the two frontal sources alone are possibly just growing in strength over time. In this analysis, shown in Figure 4B, the ERP distribution in the 500–600 ms range, a period in which only the frontal sources are presumably active, was subtracted from a later distribution between 1,100–1,200 ms, by which time our model suggests that the parietal sources have become substantially activated. If only a single frontal dipole pair were being activated in both time windows, differing solely in strength of activation between these two windows, this subtraction should reveal differential activity that also had a clearly frontal distribution, similar to that in the 500–600 ms window. If, however, the later window includes substantial activity that is generated by additional, more posterior areas, then this subtraction should yield a residual activation with a more posterior distribution on the scalp. This latter possibility is exactly the pattern that is shown in Figure 4B, in which this subtraction leaves a distribution with a posterior parietal maximum. In addition, we note that scaling of the early frontal-only activity (i.e., the distribution at 500–600 ms) up to the level of the later frontal-level amplitude (by multiplying all potentials across the scalp with the factor representing the ratio between the maximum amplitude in the two windows) before performing the subtraction from the later window revealed a very similar pattern, still leaving a clearly posterior residual distribution (Figure 4C). This analysis thus provides additional evidence of the existence of posterior sources that onset later in time, and which would thus be well explained as deriving from the parietal activations seen in the fMRI in the identical contrast.

### Pre-Target Biasing Activity in Occipital Cortex

Figure 5 shows back-view topographic plots of the activity triggered by the attention-directing cues in 200-ms bins between 500–1,900 ms post-cue, separately for attend-left and attend-right cues (two top rows). This figure suggests a relative enhancement of negative-wave activity over posterior scalp sites contralateral to the direction of attention, in addition to the large, superior, bilateral negativity reaching back to parietal sites that was discussed above. The contralaterality of this effect (relative to the direction of attention) over occipital sites can be seen more clearly in the difference waves computed from ERP responses to the attend-left–minus–attend-right cues (bottom row), with this subtraction resulting in a relative negativity over the right occipital cortex and a corresponding relative positivity over the left occipital cortex. This attend-left–minus–attend-right cue-related difference activity of contralateral negativity and ipsilateral positivity with respect to the direction of attention can be seen to begin at around 700 ms post-cue and to build up in strength over time until the onset time of the possible late target presentation. At the right side of the figure (far right column), topographic plots of a corresponding subtraction of the responses to left and right targets are displayed for the N1-latency window (corrected for overlap from preceding cue-related activity; see Methods). Comparing, it can be seen that the pattern of differential hemispheric activity elicited by the cues prior to target occurrence showed a strikingly similar scalp-potential distribution to the post-target N1 difference-wave activity. Because this activity matches the idea of preparatory activity building up over time in areas later recruited for target processing, we have termed this activity a biasing-related negativity (BRN).

**Figure 5:**
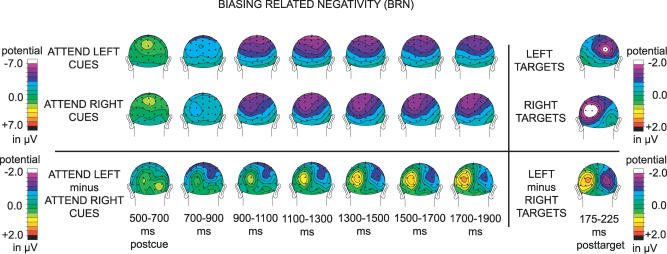
Pretarget Visual Cortex Biasing Grand-average (*n* = 13) ERP topographic plots (back view) time locked to attend-left cues (upper left row), attend-right cues (middle left row), and the difference-wave plots for the attend-left cues minus the attend-right cues (lower left row), averaged over 200-ms bins, starting 500 ms post-cue until the onset of late targets at 1,900 ms. The far right column shows N1 latency back-view topographic plots for left targets (upper right), right targets (middle right), and the left-minus-right target difference wave (lower right). All target-related activity is corrected for overlap from previous cue activity. Note the build up and then maintenance of the biasing-related negativity BRN over the occipital cortex contralateral to the direction of attention, and also note the similarity of the scalp-potential distributions of this biasing-related activity to the N1 differences between left and right targets.

The contralaterality of the BRN with respect to the cued site was confirmed by significant interactions between CUETYPE (attend-left versus attend-right cues) × HEMI (left versus right hemisphere) beginning at 900 ms post-cue and increasing across the rest of the cue-orienting period (tested in 200 ms bins of averaged data: *F*[1,12] values between 8.2–16.2, *p-*values between 0.015–0.000) over left and right parietal-occipital scalp-sites (left: O1, O3, TO1, and P3; right: O2, O4, TO2, and P4). Between 700–900 ms, the effect was only tending toward significance (*F*[1,12] = 3.8, *p* = 0.07). Finally, the same parietal-occipital scalp sites predictably revealed highly significant contralateral N1 components to the unilateral targets (TARGETTYPE × HEMI effect between 175–225 ms; *F*[1,12] = 37.58, *p* = 0.001).

## Discussion

The present study combines high-density ERP recordings with fast-rate, event-related fMRI data from an identical visual attentional-cueing paradigm to investigate the executive control of visual-spatial attention. Previous neuroimaging studies and neuropsychological lesion data had implicated a frontal-parietal network in attentional control. In addition, in our own recent fMRI attentional-cueing study [22], by using both attention-directing cues and “cue-interpretation” control trials, we had delineated a functional distinction between the lateral parts of this network as being more involved in general cue processing (such as cue interpretation), and the more medial parts as being more specific for the orienting of visual-spatial attention. In addition, the fMRI data included a reflection of pretarget biasing of the specific visual cortical areas that would be processing the upcoming target. The temporal characteristics of the activations of the various parts of this system, however, had not previously been established.

Here we report results from the corresponding ERP study, using an identical cueing paradigm as the fMRI experiment to help delineate the timing and sequence of the various parts of this network. To begin, the temporal resolution of the ERPs, in conjunction with the paradigm structure, provided a clear temporal separation between general cue processing activity, manifested as similar ERP activity until 400 ms post-cue for the attention-directing cues and interpret cues, and subsequent activity that was more specific for attentional orienting, identified as a sustained broad negativity elicited by only the attention-directing cues and lasting throughout the cue-target interval. In addition, because of the identical nature of the contrasts to those in the fMRI, the early general cue-processing ERP activity was associable with the more lateral subregions of the frontal-parietal network as delineated by the fMRI, whereas the later sustained negative wave activity for the attention-directing cues could be linked to the more medial regions of this network.

Moreover, this combined dataset allowed further parcellation of the sustained negative-wave brain activity that was specific for the attention-directing cues. In particular, both ERP traces at the scalp and multiple fMRI-constrained source modeling analyses of the ERP data indicated that the initial part of this sustained orienting-specific activity beginning at 400 ms post-cue derived primarily from the frontal cortical regions, with the parietal contribution not beginning till after 700 ms. Additional analyses further indicated that this was then followed (beginning ∼800–900 ms post-cue) by pretarget biasing activity of specific visual cortical areas contralateral to the direction of attention in preparation for the to-be-detected visual target stimulus.

### Combining fMRI and ERP Results

The model discussed above assumes that there are electrophysiological correlates of both the frontal and the parietal areas activated in the fMRI. This assumption, however, has some associated caveats. Not only do these two methods measure different aspects of brain activity (electrical field potentials during neuronal activity versus metabolic demands resulting from that neuronal activity), it is also the case that some fMRI sources might not be seen in the ERPs (e.g., activity in deep brain structures) and, conversely, that there can be circumstances in which there may not be any fMRI correlate(s) for certain ERP components of interest (e.g., very transient effects). In the current study, however, we believe that we have been able to create enough likely overlap between the possible results from the two studies to justify a direct comparison with fMRI-seeded source modeling. First, we have minimized many of the possible sources of differences between the two datasets by using exactly the same event-related paradigm and the same timing parameters in the two studies, and then mapping the activations derived from identical contrasts in the two studies. Secondly, the focus of this fMRI–ERP mapping is on the sustained attention-related preparatory activity. The electrical correlates of this activity were both quite large in amplitude (∼7 μV) and sustained for many hundreds of milliseconds, making it that much more likely to correlate well with increased metabolic demands (and thus fMRI BOLD-signal activity) in the areas of the brain involved in these processes (as compared with electrical activations of a much smaller amplitude or transient nature). It would actually be very surprising if these very large and very sustained electrical activations on the scalp did not result in an increase in metabolic and thus fMRI activity somewhere in the brain. Moreover, since there was such a robust set of fMRI activations (i.e., the frontal and parietal activations) isolated during the identical paradigm using an identical contrast, these fMRI activations seem likely to be the generating sources. Lastly, a recent study has reported that such tonic, sustained activity reflected by slow cortical potentials recorded at the scalp do seem to correlate well with an increase in metabolic demands reflected by fMRI BOLD-signal responses [54]. Thus, although there are not always one-to-one relationships between ERP and fMRI activity, in certain circumstances (such as the current one) relating the two datasets together seems quite reasonable.

Nonetheless, despite the likely correspondence between the two activation datasets, we have tested this assumption in several other ways, including an additional one not described in the Results section. In particular, another possible source-configuration model to consider for the attentional-orienting activity is that despite there being both a frontal and a parietal pair of potential sources implicated by the identical contrast in the fMRI, perhaps there is only contribution from one frontal source pair that shifts its orientation posteriorly across time, with no contribution from the parietal pair. This, however, does not seem to be very likely. To begin with, although the frontal pair alone explains the early activity extremely well (RV < 4%), an analysis in the later windows of a model using the frontal sources alone (even allowed to optimally shift in orientation) yielded a fit of the data that was considerably worse than the frontal–parietal model described above (e.g., from 1,100–1,300 ms, the RV was ∼12% versus 4%, particularly fitting poorly toward the back of the head). Even more importantly, a frontal dipole source pair that has shifted its orientation posteriorly would be expected to give rise to the spatial peak on the scalp being shifted posteriorly (indeed, our additional modeling simulations confirm this would be the case). In contrast, as can be seen in Figure 2C, this is not the observed pattern in the data; rather, the spatial peak is quite stationary during the entire interval. In actuality, the changes in the distribution of the attentional activity over time appear much more like one in which the frontal negativity expanded over time to cover the more posterior areas, while still maintaining similar, or even greater, activation over frontal areas, rather than the shifting of one activation pattern backward. This is, as we have noted, very well fit by the two-pair (frontal and parietal) model, with the parietal pair coming on line later than the frontal. Moreover, the shifting backward of the orientations of ERP sources alone does not seem very physiologically plausible unless, possibly, if it were due to the coming on line later of differential portions within the frontal sources that happened to point backward. However, considering the clear and robust posterior (i.e., parietal activations) seen in the fMRI in an identical contrast, this shifting hypothesis, besides not fitting the distributions nearly as well, seems considerably less plausible than our model in which the source of this increased posterior distribution at longer latencies is actually due to the later onsetting of contribution from these parietal sources.

In addition, various consideration and analyses of the ERP data alone, without explicitly incorporating the fMRI, also implicate a match to the frontal and parietal fMRI activations and indicate the onset of frontal activity prior to a parietal source. For example, Figure 2C shows a clear distribution spread over time, including first only frontal cortical areas, and then later over central and then parietal areas. In addition, the ERP overlays of frontal and parietal recording sites (see Figure 3A) also clearly point to parietal activity starting later than frontal activity. And last but not least, Figure 4B and 4C show that when the early activity (either unscaled or scaled) is subtracted from the later activity on the scalp, it leaves a clearly posterior distribution of activity over parietal scalp. All these aspects of the ERP data alone are very suggestive of an onset at 400 ms of orienting-related activity in the frontal cortex, followed a few hundred milliseconds later by the addition of parietal cortex activity, thus convergent with the ERP source analyses seeded by the corresponding fMRI activity foci.

Thus, these data suggest the following sequence of functional activity in response to an attention-directing instructional cue. In the first 400 ms or so, there is general cue processing, including cue sensory processing (in visual cortex) and cue-symbol interpretation (largely in lateral regions of the frontal–parietal network). After this, the more medial portions of the frontal cortex initiate activity that is more specific for the orienting of attention, followed a couple hundred milliseconds later by medial parietal activity. This relative latency delay for the medial parietal onset suggests that the initial medial frontal activity for attentional orienting may signal the parietal regions to activate, which may in turn help facilitate the specific biasing activity in visual cortex shortly after. Lastly, our data also suggest that after activation initiation, both the medial frontal and medial parietal regions appear to maintain sustained levels of activity throughout the cue-target interval. This suggests that although there is different onset timing of their sustained activations, these frontal and parietal regions presumably continue to work together in maintaining the appropriate attentional state and resulting target-location specific preparation of relevant sensory areas.

### Functional Interpretation of the Results

The critical role of the frontal cortex in the activation pattern in the present study fits well with some current theories concerning its central role in executive and attentional control. In particular, frontal regions have been described as being involved in the following tasks: (1) keeping track of task goals and actively maintaining a representation of stimulus-response associations [55]; (2) controlling temporal aspects of the task, such as linking past sensory memory traces to future (motor) goals [56]; (3) expressing preparation based on hypotheses about most likely identity and task relevancy of the upcoming stimulus [57]; (4) regulating selection of task relevant stimuli and responses in an attentional set [16]; and (5) controlling sustained attentional activity in posterior areas that express attentional preparation [58]. In the context of these theoretical notions, our study suggests that the more lateral frontal regions, along with the lateral parietal ones, play a role in more general executive processes. In the present study, these more-general executive processes would appear to include activity in the first 400 ms after a symbolic instructional cue that involves interpreting the meaning of that cue, and making a decision as to what to do based on that interpretation. These are processes that needed to be performed for both the attend cues and the interpret cues. Moreover, the present results suggest that after these initial, more-general executive processes, the medial frontal regions then specifically perform regulatory control over the initiation and maintenance of the orienting of visual-spatial attention. The temporal characteristics of the activations suggest that this attentional control may involve these medial frontal regions signaling or triggering the parietal regions to activate to facilitate the biasing of visual areas to enhance the processing of expected goal-relevant stimuli. Thus, this activation pattern for the medial frontal regions supports its role in keeping track of task goals and in controlling and coordinating other regions to help accomplish those goals [55].

Functional interpretation of the role of parietal cortex in attentional control has been described as contributing to the following: (1) the generation of spatial stimulus-response mapping patterns of activity [59], (2) the establishment of or switching of task-sets or spatial stimulus-response associations [16,60], (3) the rehearsal of to-be-memorized locations in spatial working memory [61], and (4) the modulation of neuronal activity in the visual cortex [62]. As noted above, the present results suggest that the more lateral parietal subregions, together with the lateral frontal subregions, are involved in the initial cue-symbol interpretation and decisional processes based on that interpretation. Following these processes, and then following activation of the medial frontal regions that appear to initiate the specific orienting of attention toward the task-relevant stimuli/location, the medial parietal regions would appear to activate and stay active throughout the cue-target period, presumably participating in the triggering of the biasing seen shortly after in visual sensory cortex.

With respect to the baseline shift in target-location specific visual areas, this mechanism of attentional control over expected upcoming processing of task-relevant stimuli has been linked in previous ERP studies to a component termed a late-directing attention positivity (LDAP) [23–25]. Our observed biasing-related activity over occipital-parietal cortex (BRN), however, differs from these previous findings in a number of ways. First, this BRN consists of a pattern of increased negativity over occipital areas that are contralateral to the direction of attention, whereas the LDAP shows the opposite pattern (contralateral positivity). Second, the BRN steadily increased in strength across the cue-target interval and continued its highest level of activity until target onset, and even beyond, when no target is presented (i.e., in cue-only trials). The LDAP, in contrast, has been described by some researchers to be more transient in nature and to disappear shortly before target onset [25]. Third, an LDAP-like component has been found to be elicited not only during visual-spatial orienting, but also when attention is directed towards expected locations of auditory or tactile events [63], suggesting that such a component may reflect a more general aspect of target preparation than just the modulation of baseline activity in target-location–specific visual-sensory areas. Finally, the onset latencies of the BRN observed here and previously reported LDAP responses differ in that the BRN appears to need more time to build up than the LDAP. Even though the target could appear moderately early (900 ms), the BRN did not begin till around 800–900 ms post-cue, whereas the LDAP has mostly been observed in an earlier window between approximately 500–700 ms post-cue.

The discrepancies between our BRN and the LDAP component might be related to different design parameters used in the present study compared to some previous ERP studies, such as somewhat longer delays between cues and targets, the use of only validly cued target locations, and the use of delayed rather than speeded responses to targets. Alternatively, these two components might reflect different underlying mechanisms of target-specific biasing activity. For example, the LDAP or earlier-latency lateralized activity could reflect processing related to the establishment of stimulus-response mapping representations [63,64], whereas the BRN might reflect the specific sustained baseline shift that biases target-specific brain areas to enhance perceptual sensitivity, and that is maintained across the attentional-orienting time period. Lastly, the relatively late start of the biasing in the present study could be related to the specific manipulation of probability and timing of target occurrence in the present study (moderately early or late, sometimes not presented), as suggested recently by Correa and colleagues [65]. More specifically, for example, had we included trials in which the target could have come earlier (e.g., by 400–800 ms post-cue), both the attentional-orienting control activity and the biasing activity may well have begun earlier.

In conclusions, our results provide evidence for different functional roles of the various brain areas involved in visual-spatial attentional orienting by revealing differential temporal characteristics during the cue-target interval. First, the combined pattern of ERP and fMRI data suggest that the initial processing of the cue in the first 350–400 ms involves the analysis of its sensory content and the decoding of its instructional meaning. These processes are presumably performed in visual cortex, and then in lateral frontal and parietal areas, respectively, and are carried out even when the cue instructs to not orient attention (e.g., the interpret-cue control trials in this study). Second, after this initial general cue processing, the more medial frontal areas initiate attentional orienting, including triggering the onset and maintenance of medial parietal cortex activation. These areas in turn have a role in rendering a biasing of activity in those specific sensory areas that will later receive incoming signals from target stimuli. Together they prepare the system to successfully identify hard-to-detect targets. Future research should assess whether this model of visual-spatial attentional control can be generalized to other attentional-control processes and/or to more complex task situations.

## Materials and Methods

### Participants.

Sixteen healthy individuals with normal or corrected-to-normal vision gave written informed consent, approved by the Duke University Institutional Review Board, and participated in this experiment. Thirteen participants (four females and nine males, mean age 22 y, range 18–41 y, all right- handed) were included in the final analyses. Data from the other three participants were excluded from the analysis due to more than 30% of their trials being contaminated with either eye blinks, eye movements, muscle activity, or excessive drift of scalp potentials. Participants were either paid $10/hour or received university class credits for their participation.

### Design.

As in our corresponding previous fMRI study [22], participants were presented with a series of event trials, each beginning with an instructional letter cue at fixation (center of the screen), which was sometimes followed by a target. These instructional cues were either the letter “L”, “R”, or “P”, which instructed the participant either to covertly attend to a boxed location in the lower left (“L”) or lower right (“R”) visual field (3° lateral and 3° below horizontal meridian), or to not orient attention away from fixation (“P”). Targets consisted of a small faint gray dot presented on a black background in the lower visual field box on the cued side, which could occur in one or the other of two possible corners of the box (closest or farthest away from fixation). The dots differed in level of grayness ranging from 10%–19% gray (0% being equal to the completely black background), thus ranging in difficulty from trial to trial. For each target-containing trial, the target was chosen randomly from across this range to ensure that the participants could not predict the difficulty level and therefore needed to prepare maximally on each trial. Participants received at least two practice runs of 64 trials each (total run time of 4 min 48 s per run) to familiarize themselves with the task, followed by 12–14 runs (also 64 trials each) during which EEGs were recorded.

The trial structure used in this ERP study was identical to the one used in the earlier analogous fMRI study [22]. In attend trials (“L” or “R” cues), participants were instructed to determine whether a faint visual target (dot) occurred at the cued location. In attend cue–plus-target trials (25% of all trials), a target would occur either early (50% probability) at 900 ms, or late (50% probability) at 1,900 ms after cue onset. In attend-cue–only trials (25% of all trials), no target was presented. On interpret-cue trials (“P” cues; 25% of all trials), it was also the case that no faint dot target would occur. In addition, as in the fMRI study, 25% “NoStim” trials (periods of fixation only) were included, randomized with the other trials types, to facilitate the removal of response overlap from previous trials [66–68].

In all trial types (other than NoStims), an end-of-trial stimulus (the letters REP) was presented below fixation and midway between the upper part of the outlined target-boxes 2,700 ms after trial onset (Figure 1). For attend-cue trials (“L” and “R” cues), participants were instructed to press a button with their right index finger if they had observed a target in that trial. In order to minimize motor preparation within the cue-target interval on these trials, participants were explicitly instructed not to prepare for any response during the cue-target interval and to wait until the onset of the REP/end-of-trial signal to respond. The end-of-trial stimulus was, however, presented in all cue-only and cue-plus-target trials (including interpret trials) to equate sensory processing demands across conditions. Finally, trial onset-to-onset intervals were 4,500 ms for all possible trial types, including NoStims.

### ERP procedures and analyses.

The EEG was recorded from 64 electrodes mounted in a custom-designed electrocap (Electro-Cap International; http://www.electro-cap.com) and referenced to the right mastoid during recording. The 64 channels of these caps were equally spaced across the cap and covered the whole head from above the eyebrows to lower aspects of the occipital lobe (slightly past the inion). In previous work [5,69], further refined and confirmed here, the average electrode positions of this cap were determined in Talairach space, facilitating source modeling using and/or comparing with the activations from the fMRI study.

Eye blinks and eye movements were monitored by horizontal and vertical electro-oculogram (EOG) electrodes for later rejection of trials with such artifacts. Vertical eye movements and eye blinks were detected by two electrodes placed below the orbital ridge of each eye, each referenced to the electrodes above the eye. In addition, participants were encouraged to delay their blinks to the window following the report signal (2,700 ms after the cue). Horizontal eye movements were monitored by two electrodes placed at the outer canthi of the eyes. Participants were trained before starting the experiment on being able to covertly move their attention without moving their eyes. In addition, during recordings of their EEG data, eye movements were monitored by using a video zoom lens camera. Analyses of the horizontal EOG data indicated that the number of rejected trials due to eye movements was very low in all conditions (∼3%) and did not significantly differ between the different conditions. Electrode impedances were maintained below 2 kΩ for the mastoids, below 10 kΩ for the facial electrodes, and below 5 kΩ for all remaining electrodes. All 64 EEG channels were continuously recorded with a band-pass filter of 0.01–100 Hz and a gain of 1,000 (SynAmps amplifiers from Neuroscan; http://www.neuroscan.com) and digitized with a sampling rate of 500 Hz. Recordings took place in an electrically shielded, sound-attenuated, dimly lit experimental chamber.

Behavioral data of each participant were monitored and analyzed online using a custom in-house behavioral monitoring and analysis system. The output of these analyses was used during the experiment to continuously titrate task difficulty (by adjusting perceptual contrast of the targets) to keep the participant's behavior at the same level (∼80% hit rate) as in our earlier corresponding fMRI study. Target reaction times were also monitored online, although due to the delayed response, such reaction time information mainly just indicated that the participant was reasonably engaged in the task.

ERPs time-locked to the cues were averaged separately for interpret-cue and attend-cue trials with an epoch-length of 3,200 ms (including 400 ms pre-stimulus baseline). To increase signal-to-noise ratios for the cue response, cue-only trials were averaged together with cue–followed-by–late-target trials, separately for attend-right and attend-left cues as well as collapsed over both the attend-right and attend-left cues. Trials in which targets followed cue presentation early (i.e., at 900 ms) were not included in these response averages, so that the whole 1,900-ms cue-target interval could be analyzed for cue-triggered activity. It should be noted, however, that the possibility of having a target occur at both early and late time periods ensured that participants needed to process the cue and orient their attention as soon as possible and needed to maintain that attention throughout the interval. Artifact rejection was performed off-line before averaging by discarding epochs of the EEG that were contaminated by eye movements, eye blinks, excessive muscle-related potentials, drifts, or amplifier blocking when these artifacts were detected in the window of interest (−200 until 1,900 ms post-cue). Furthermore, the averages were digitally low-pass filtered with a noncausal, zero-phase, running-average filter of nine points, which strongly reduces frequencies at and above 56 Hz at our sampling frequency of 500 Hz. Additional processing of the data included re-referencing of all channels to the algebraic mean of the two mastoid electrodes. To assess orienting-specific activity, difference waves were computed for “attend-minus-interpret cues” (attend-cue–only trials collapsed with attend-cue–followed by–late-target trials, minus interpret-cue trials). Finally, spherical-spline–interpolated topographic voltage maps [70] of grand-averaged ERP traces (*n* = 13 participants) were derived for a series of consecutive time windows to visualize scalp distribution changes over time.

ERP averages time locked to early and late targets were calculated, separately for the right and left target conditions, along with analogous averages for early and late “no-target” trials (analogous points in time on cue-only trials where a target could be expected to occur but did not appear). These “no-target” trials were subsequently subtracted from the corresponding target trials in order to correct for overlap of cue-induced attentional-orienting activity [71], which was expected to differ for attend-left and attend-right cues. If not corrected for, the differential overlap for the attend-left and attend-right cues would have confounded the target-induced ERP responses. In order to increase signal-to-noise ratios, after the correction for possible overlap, early- and late-target ERPs were averaged together, separately for right and left targets. Finally, these overlap-corrected right (early + late)-target–evoked ERPs were subtracted from overlap-corrected left (early + late)-target–evoked ERPs to extract target-induced contralaterality of response to be able to compare its distribution to any cue-induced pretarget priming or biasing.

Statistical analysis for the cue-induced attentional-orienting effects (frontal–parietal network) included within-subject repeated-measures ANOVAs of the ERP amplitudes using the factor ATTENTION (with attend-right and attend-left cue-only and cue–followed-by–late-target trials collapsed together versus interpret-cue trials). Separate ANOVAs were computed for data averaged over all sample points within sequential windows of 100 ms post-cue and separately for each electrode site. To correct for multiple comparisons, the significance-level threshold (*p-*value) was lowered to 0.01 (corresponding *F*[1,12] = 9.30).

Biasing activity over the occipital cortex that was evoked by the cues (i.e., the late cue-induced BRN; see Results) was tested with repeated-measures ANOVAs on mean ERP amplitudes in 200-ms windows between 500–1,900 ms post-cue, using the factors CUETYPE (attend-left cues versus attend-right cues) and HEMI (left versus right hemisphere). Electrodes included in these ANOVAs were restricted to four left parietal-occipital sites (O1, O3, TO1, and P3) and four right parietal-occipital sites (O2, O4, TO2, and P4). Because of the very specific planned comparisons used in this analysis, significance was assumed for *F*[1,12] values larger than 4.7, corresponding to a *p-*value of 0.05. The same electrode sites were used in repeated-measures ANOVAs of the ERP amplitudes of the right- and left-target trials (collapsed across early and late), to test contralaterality of the N1 component between 175–225 ms post targets (factors included TARGETTYPE [right versus left targets] and HEMI [left versus right hemisphere]).

Finally, ERP dipole source analysis—using the BESA software (Brain Electrical Source Analysis, Version 2; http://www.besa.de)—was performed on the attend-minus-interpret cue grand-average difference waves (*n* = 13), which predominantly reflect attentional-orienting processes. In this study, we were able to constrain the number and possible source locations by using activated loci found within the identical contrast in our previous corresponding fMRI study. The fMRI locations were seeded into BESA, using their Talairach locations and the Talairach-to-spherical transformation parameters from our cap electrodes. The default four-shell, spherical head model was used in modeling of those sources. Allowing orientation to vary, the seeded sources were fit in the latency window of statistically significant attentional-orienting activity, keeping their locations constrained to the fMRI-locations. An energy criterion of 50% was used, which helps minimize interactions during the iterative modeling procedure between sources that are relatively close to one another. The source analyses were applied to both the grand-average (*n* = 13 participants) and the single-subject attentional-orienting difference wave activity. The single-subject analyses were accompanied by statistical analyses of the dipole strengths over time (averaged over 100 ms of data and over hemisphere) of the estimated source activity.

Lastly, early cue-induced attentional-orienting activity (500–600 ms) was modeled in a second “free-source-modeling” approach, using a single symmetrical dipole pair, optimizing both location and orientation. Final solutions of this analysis were transformed back into Talairach space and compared to the fMRI-seeded sources.

## Abbreviations

ANOVA: analysis of variance
BOLD: blood-oxygen-level–dependent
BRN: biasing-related negativity
EEG: electroencephalography
EOG: electrooculogram
ERP: event-related potential
fMRI: functional magnetic resonance imaging
LDAP: late-directing attention positivity
RV: residual variance
SOA: stimulus onset asynchrony
ISI: interstimulus interval

## References

[pbio-0050012-b001] Posner MI, Snyder CR, Davidson BJ (1980). Attention and the detection of signals. J Exp Psychol.

[pbio-0050012-b002] Downing CJ (1988). Expectancy and visual-spatial attention: Effects on perceptual quality. J Exp Psychol Hum Percept Perform.

[pbio-0050012-b003] Mangun GR, Hillyard SA (1991). Modulations of sensory-evoked brain potentials indicate changes in perceptual processing during visual-spatial priming. J Exp Psychol Hum Percept Perform.

[pbio-0050012-b004] Heinze HJ, Mangun GR, Burchert W, Hinrichs H, Scholz M (1994). Combined spatial and temporal imaging of brain activity during visual selective attention in humans. Nature.

[pbio-0050012-b005] Woldorff MG, Fox PT, Matzke M, Lancaster JL, Veeraswamy S (1997). Retinotopic organization of early visual spatial attention effects as revealed by PET and ERPs. Human Brain Mapping.

[pbio-0050012-b006] Mangun GR, Buonocore MH, Girelli M, Jha AP (1998). ERP and fMRI measures of visual spatial selective attention. Hum Brain Mapp.

[pbio-0050012-b007] Hopfinger JB, Buonocore MH, Mangun GR (2000). The neural mechanisms of top-down attentional control. Nat Neurosci.

[pbio-0050012-b008] Handy TC, Green V, Klein RM, Mangun GR (2001). Combined expectancies: Event-related potentials reveal the early benefits of spatial attention that are obscured by reaction time measures. J Exp Psychol Hum Percept Perform.

[pbio-0050012-b009] Corbetta M (1998). Frontoparietal cortical networks for directing attention and the eye to visual locations: Identical, independent, or overlapping neural systems?. Proc Natl Acad Sci U S A.

[pbio-0050012-b010] Corbetta M, Miezin FM, Shulman GL, Petersen SE (1993). A PET study of visuospatial attention. J Neurosci.

[pbio-0050012-b011] Giesbrecht B, Woldorff MG, Song AW, Mangun GR (2003). Neural mechanisms of top-down control during spatial and feature attention. Neuroimage.

[pbio-0050012-b012] Gitelman DR, Nobre AC, Parrish TB, LaBar KS, Kim YH (1999). A large-scale distributed network for covert spatial attention: Further anatomical delineation based on stringent behavioural and cognitive controls. Brain.

[pbio-0050012-b013] Kastner S, De Weerd P, Desimone R, Ungerleider LG (1998). Mechanisms of directed attention in the human extrastriate cortex as revealed by functional MRI. Science.

[pbio-0050012-b014] Nobre AC, Sebestyen GN, Gitelman DR, Mesulam MM, Frackowiak RS (1997). Functional localization of the system for visuospatial attention using positron emission tomography. Brain.

[pbio-0050012-b015] Vandenberghe R, Gitelman DR, Parrish TB, Mesulam MM (2001). Location- or feature-based targeting of peripheral attention. Neuroimage.

[pbio-0050012-b016] Corbetta M, Shulman GL (2002). Control of goal-directed and stimulus-driven attention in the brain. Nat Rev Neurosci.

[pbio-0050012-b017] Chelazzi L, Miller EK, Duncan J, Desimone R (1993). A neural basis for visual search in inferior temporal cortex. Nature.

[pbio-0050012-b018] Fu KM, Foxe JJ, Murray MM, Higgins BA, Javitt DC (2001). Attention-dependent suppression of distracter visual input can be cross-modally cued as indexed by anticipatory parieto-occipital alpha-band oscillations. Brain Res Cogn Brain Res.

[pbio-0050012-b019] Kastner S, Pinsk MA, De Weerd P, Desimone R, Ungerleider LG (1999). Increased activity in human visual cortex during directed attention in the absence of visual stimulation. Neuron.

[pbio-0050012-b020] Luck SJ, Chelazzi L, Hillyard SA, Desimone R (1997). Neural mechanisms of spatial selective attention in areas V1, V2, and V4 of macaque visual cortex. J Neurophysiol.

[pbio-0050012-b021] Reynolds JH, Chelazzi L, Desimone R (1999). Competitive mechanisms subserve attention in macaque areas V2 and V4. J Neurosci.

[pbio-0050012-b022] Woldorff MG, Hazlett CJ, Fichtenholtz HM, Weissman DH, Dale AM (2004). Functional parcellation of attentional control regions of the brain. J Cogn Neurosci.

[pbio-0050012-b023] Harter MR, Anllo-Vento L (1991). Visual-spatial attention: Preparation and selection in children and adults. Electroencephalogr Clin Neurophysiol Suppl.

[pbio-0050012-b024] Yamaguchi S, Tsuchiya H, Kobayashi S (1994). Electroencephalographic activity associated with shifts of visuospatial attention. Brain.

[pbio-0050012-b025] Hopf JM, Mangun GR (2000). Shifting visual attention in space: An electrophysiological analysis using high spatial resolution mapping. Clin Neurophysiol.

[pbio-0050012-b026] Nobre AC, Sebestyen GN, Miniussi C (2000). The dynamics of shifting visuospatial attention revealed by event-related potentials. Neuropsychologia.

[pbio-0050012-b027] Slagter HA, Kok A, Mol N, Kenemans JL (2005). Spatio-temporal dynamics of top-down control: Directing attention to location and/or color as revealed by ERPs and source modeling. Brain Res Cogn Brain Res.

[pbio-0050012-b028] Talsma D, Slagter HA, Nieuwenhuis S, Hage J, Kok A (2005). The orienting of visuospatial attention: An event-related brain potential study. Brain Res Cogn Brain Res.

[pbio-0050012-b029] Logothetis NK, Pauls J, Augath M, Trinath T, Oeltermann A (2001). Neurophysiological investigation of the basis of the fMRI signal. Nature.

[pbio-0050012-b030] Di Russo F, Martinez A, Hillyard SA (2003). Source analysis of event-related cortical activity during visuo-spatial attention. Cereb Cortex.

[pbio-0050012-b031] Martinez A, DiRusso F, Anllo-Vento L, Sereno MI, Buxton RB (2001). Putting spatial attention on the map: Timing and localization of stimulus selection processes in striate and extrastriate visual areas. Vision Res.

[pbio-0050012-b032] Noesselt T, Hillyard SA, Woldorff MG, Schoenfeld A, Hagner T (2002). Delayed striate cortical activation during spatial attention. Neuron.

[pbio-0050012-b033] Horovitz SG, Rossion B, Skudlarski P, Gore JC (2004). Parametric design and correlational analyses help integrating fMRI and electrophysiological data during face processing. Neuroimage.

[pbio-0050012-b034] Henson RN, Goshen-Gottstein Y, Ganel T, Otten LJ, Quayle A (2003). Electrophysiological and haemodynamic correlates of face perception, recognition and priming. Cereb Cortex.

[pbio-0050012-b035] Swainson R, Cunnington R, Jackson GM, Rorden C, Peters AM (2003). Cognitive control mechanisms revealed by ERP and fMRI: Evidence from repeated task-switching. J Cogn Neurosci.

[pbio-0050012-b036] Iidaka T, Matsumoto A, Haneda K, Okada T, Sadato N (2006). Hemodynamic and electrophysiological relationship involved in human face processing: Evidence from a combined fMRI-ERP study. Brain Cogn.

[pbio-0050012-b037] Mathalon DH, Whitfield SL, Ford JM (2003). Anatomy of an error: ERP and fMRI. Biol Psychol.

[pbio-0050012-b038] Ullsperger M, von Cramon DY (2001). Subprocesses of performance monitoring: A dissociation of error processing and response competition revealed by event-related fMRI and ERPs. Neuroimage.

[pbio-0050012-b039] Doeller CF, Opitz B, Mecklinger A, Krick C, Reith W (2003). Prefrontal cortex involvement in preattentive auditory deviance detection: Neuroimaging and electrophysiological evidence. Neuroimage.

[pbio-0050012-b040] Mulert C, Jager L, Propp S, Karch S, Stormann S (2005). Sound level dependence of the primary auditory cortex: Simultaneous measurement with 61-channel EEG and fMRI. Neuroimage.

[pbio-0050012-b041] Liebenthal E, Ellingson ML, Spanaki MV, Prieto TE, Ropella KM (2003). Simultaneous ERP and fMRI of the auditory cortex in a passive oddball paradigm. Neuroimage.

[pbio-0050012-b042] Opitz B, Mecklinger A, von Cramon DY, Kruggel F (1999). Combining electrophysiological and hemodynamic measures of the auditory oddball. Psychophysiology.

[pbio-0050012-b043] Opitz B, Rinne T, Mecklinger A, von Cramon DY, Schroger E (2002). Differential contribution of frontal and temporal cortices to auditory change detection: fMRI and ERP results. Neuroimage.

[pbio-0050012-b044] Menon V, Ford JM, Lim KO, Glover GH, Pfefferbaum A (1997). Combined event-related fMRI and EEG evidence for temporal-parietal cortex activation during target detection. Neuroreport.

[pbio-0050012-b045] Bledowski C, Prvulovic D, Hoechstetter K, Scherg M, Wibral M (2004). Localizing P300 generators in visual target and distractor processing: A combined event-related potential and functional magnetic resonance imaging study. J Neurosci.

[pbio-0050012-b046] Bledowski C, Prvulovic D, Goebel R, Zanella FE, Linden DE (2004). Attentional systems in target and distractor processing: A combined ERP and fMRI study. Neuroimage.

[pbio-0050012-b047] Ford JM, Gray M, Whitfield SL, Turken AU, Glover G (2004). Acquiring and inhibiting prepotent responses in schizophrenia: Event-related brain potentials and functional magnetic resonance imaging. Arch Gen Psychiatry.

[pbio-0050012-b048] Rowan A, Liegeois F, Vargha-Khadem F, Gadian D, Connelly A (2004). Cortical lateralization during verb generation: A combined ERP and fMRI study. Neuroimage.

[pbio-0050012-b049] Matsumoto A, Iidaka T, Haneda K, Okada T, Sadato N (2005). Linking semantic priming effect in functional MRI and event-related potentials. Neuroimage.

[pbio-0050012-b050] Rossell SL, Price CJ, Nobre AC (2003). The anatomy and time course of semantic priming investigated by fMRI and ERPs. Neuropsychologia.

[pbio-0050012-b051] Nagai Y, Critchley HD, Featherstone E, Fenwick PB, Trimble MR (2004). Brain activity relating to the contingent negative variation: An fMRI investigation. Neuroimage.

[pbio-0050012-b052] Hinterberger T, Veit R, Strehl U, Trevorrow T, Erb M (2003). Brain areas activated in fMRI during self-regulation of slow cortical potentials (SCPs). Exp Brain Res.

[pbio-0050012-b053] Talairach J, Tournoux P (1988). Co-planar stereotaxic atlas of the human brain.

[pbio-0050012-b054] Schicke T, Muckli L, Beer AL, Wibral M, Singer W (2006). Tight covariation of BOLD signal changes and slow ERPs in the parietal cortex in a parametric spatial imagery task with haptic acquisition. Eur J Neurosci.

[pbio-0050012-b055] Miller EK, Cohen JD (2001). An integrative theory of prefrontal cortex function. Annu Rev Neurosci.

[pbio-0050012-b056] Fuster JM (2001). The prefrontal cortex–an update: Time is of the essence. Neuron.

[pbio-0050012-b057] Ivry R, Knight RT (2002). Making order from chaos: The misguided frontal lobe. Nat Neurosci.

[pbio-0050012-b058] LaBerge D (2002). Attentional control: Brief and prolonged. Psychol Res.

[pbio-0050012-b059] Colby CL, Goldberg ME (1999). Space and attention in parietal cortex. Annu Rev Neurosci.

[pbio-0050012-b060] Yantis S, Schwarzbach J, Serences JT, Carlson RL, Steinmetz MA (2002). Transient neural activity in human parietal cortex during spatial attention shifts. Nat Neurosci.

[pbio-0050012-b061] Postle BR, Awh E, Jonides J, Smith EE, D'Esposito M (2004). The where and how of attention-based rehearsal in spatial working memory. Brain Res Cogn Brain Res.

[pbio-0050012-b062] Han S, Jiang Y, Gu H, Rao H, Mao L (2004). The role of human parietal cortex in attention networks. Brain.

[pbio-0050012-b063] Eimer M, van Velzen J, Driver J (2002). Cross-modal interactions between audition, touch, and vision in endogenous spatial attention: ERP evidence on preparatory states and sensory modulations. J Cogn Neurosci.

[pbio-0050012-b064] Praamstra P, Boutsen L, Humphreys GW (2005). Frontoparietal control of spatial attention and motor intention in human EEG. J Neurophysiol.

[pbio-0050012-b065] Correa A, Lupianez J, Tudela P (2006). The attentional mechanism of temporal orienting: Determinants and attributes. Exp Brain Res.

[pbio-0050012-b066] Burock MA, Buckner RL, Woldorff MG, Rosen BR, Dale AM (1998). Randomized event-related experimental designs allow for extremely rapid presentation rates using functional MRI. Neuroreport.

[pbio-0050012-b067] Buckner RL (1998). Event-related fMRI and the hemodynamic response. Hum Brain Mapp.

[pbio-0050012-b068] Busse L, Woldorff MG (2003). The ERP omitted stimulus response to “no-stim” events and its implications for fast-rate event-related fMRI designs. Neuroimage.

[pbio-0050012-b069] Woldorff MG, Liotti M, Seabolt M, Busse L, Lancaster JL (2002). The temporal dynamics of the effects in occipital cortex of visual-spatial selective attention. Brain Res Cogn Brain Res.

[pbio-0050012-b070] Perrin F, Pernier J, Bertrand O, Echallier JF (1989). Spherical splines for scalp potential and current density mapping. Electroencephalogr Clin Neurophysiol.

[pbio-0050012-b071] Woldorff MG (1993). Distortion of ERP averages due to overlap from temporally adjacent ERPS - analysis and correction. Psychophysiology.

